# Computational Identification of Master Regulators Influencing Trypanotolerance in Cattle

**DOI:** 10.3390/ijms22020562

**Published:** 2021-01-08

**Authors:** Abirami Rajavel, Armin Otto Schmitt, Mehmet Gültas

**Affiliations:** 1 Breeding Informatics Group, Department of Animal Sciences, Georg-August University, Margarethe von Wrangell-Weg 7, 37075 Göttingen, Germany; abirami.rajavel@uni-goettingen.de (A.R.); armin.schmitt@uni-goettingen.de (A.O.S.); 2 Center for Integrated Breeding Research (CiBreed), Albrecht-Thaer-Weg 3, Georg-August University, 37075 Göttingen, Germany

**Keywords:** animal african trypanosomiasis, master regulators, over-represented pathways, *DBP*, *MYC*

## Abstract

African Animal Trypanosomiasis (AAT) is transmitted by the tsetse fly which carries pathogenic trypanosomes in its saliva, thus causing debilitating infection to livestock health. As the disease advances, a multistage progression process is observed based on the progressive clinical signs displayed in the host’s body. Investigation of genes expressed with regular monotonic patterns (known as Monotonically Expressed Genes (MEGs)) and of their master regulators can provide important clue for the understanding of the molecular mechanisms underlying the AAT disease. For this purpose, we analysed MEGs for three tissues (liver, spleen and lymph node) of two cattle breeds, namely trypanosusceptible Boran and trypanotolerant N’Dama. Our analysis revealed cattle breed-specific master regulators which are highly related to distinguish the genetic programs in both cattle breeds. Especially the master regulators *MYC* and *DBP* found in this study, seem to influence the immune responses strongly, thereby susceptibility and trypanotolerance of Boran and N’Dama respectively. Furthermore, our pathway analysis also bolsters the crucial roles of these master regulators. Taken together, our findings provide novel insights into breed-specific master regulators which orchestrate the regulatory cascades influencing the level of trypanotolerance in cattle breeds and thus could be promising drug targets for future therapeutic interventions.

## 1. Introduction

African Animal Trypanosomiasis (AAT), also known as ’nagana’, is a parasitic disease of animals caused by the flagellated protozoan species of *Trypanosoma* which is transmitted primarily through the bite of infected tsetse flies [1]. This neglected disease is a threat to animal and human health, especially in sub-Saharan African countries [2,3,4]. It affects millions of livestock annually, leading to major economic loss of billions of US dollars every year and substantial decrease in agricultural productivity in Africa [5,6].

*Trypanosoma congolense*, *Trypanosoma vivax*, and *Trypanosoma brucei* are the major causes of infection in livestock [7]. Trypanotolerance refers to the capability of the animal to control parasitaemia and anaemia and to remain productive despite the infection of the parasite [8,9,10]. It has become an important trait in the recent decade and this trait is widely found in some *Bos taurus* cattle breeds including N’Dama and West African shorthorn breeds [9,11,12,13]. Although the aforementioned cattle breeds remain productive during the course of the disease, they are not desirable for farming due to their smaller size and lower draft power. On the other hand, Zebu (*Bos indicus*) cattle are not particularly resistant to trypanosomiasis, unlike the native *Bos taurus* cattle. Farmers depend on the Zebu cattle breeds like Boran as they are more suitable for agricultural purposes due to their high draught power and agricultural productivity. However, susceptibility of these breeds to trypanosomiasis poses heavy economic constraints to the farmers [9,10,14,15,16,17,18,19].

Until now, several studies have been performed in cattle by analysing genotype or gene expression data in order to understand the molecular mechanism underlying the genetic resistance to African trypanosomiasis [20,21,22,23,24,25]. Hanotte et al. [25] identified quantitative trait loci (QTL) controlling trypanotolerance in a cross of tolerant West African N’Dama and susceptible East African Boran cattle. Moreover, Mekonnen et al. [20] surveyed the genome of the cattle breed Sheko to study the genotype-phentotype associations and identified genomic regions associated with trypanosomiasis. On the other hand, O’Gorman et al. [22] identified temporal changes in peripheral blood mononuclear cell (PBMC) gene expression in trypanotolerant N’Dama and trypanosusceptible Boran, by studying transcriptomic profiles during the disease progression. To this end, Noyes et al. [26] performed gene expression analysis and identified several candidate genes in pathways which responded to trypanosome infection in Boran and N’Dama.

Recently, by analysing the gene expression data set generated by Noyes et al. [26], we have deciphered the cattle breed-specific partner choice of transcription factors (TFs) during the disease progression [27]. For this purpose, we mainly considered the Monotonically Expressed Genes (MEGs) to capture the multistage progression process of the AAT disease in liver, spleen and lymph node tissues. Importantly, we highlighted the pivotal relevance of the preferential partner choice of the TF albumin D-site-Binding Protein (*DBP*) in these tissues.

It has been widely shown that the transcriptional regulation of *DBP* controls the circadian output/behaviour not only in the suprachiasmatic nucleus SCN [28], but also in the peripheral tissues [29,30]. Taking this aspect into account, we addressed the association of *DBP* with circadian transcriptional regulation of tissue-specific processes in mammalian species, in the context of AAT. Especially our findings implicating the functional relationship of circadian control with the immune system are well substantiated by Solis et al. [31], Barik et al. [32] and Scheiermann et al. [33]. In reference to the study by Frank Hawking [34], in which he experimentally established the link between circadian rhythm and *T. congolense* infection in the blood of laboratory rodents, our findings also support the important role of the circadian rhythm in the AAT disease.

Exploring tissue-specific regulatory mechanisms is of utmost importance, especially in tissues such as liver, spleen and lymph nodes, which are likely the primary sites where anaemia occurs as extra-vascular haemolysis [13,35,36]. Therefore, similar to our previous studies [37,38], taking advantage of the systems biology approaches, we attempted in this study to gain novel insights by unravelling the tissue- and breed-specific master regulators and over-represented signalling pathways that responded to trypanosome infection in Boran and N’Dama. Surprisingly, our results show *DBP* as a master regulator for liver tissue of N’Dama, emphasizing the role of the circadian rhythm in the hepatic metabolism and in the immune responses after trypanosome infection in this resistant breed. Altogether, our results highlight a striking feature of the circadian clock in trypanotolerance, especially in the regulatory role of *DBP* in the immunity of trypanotolerant cattle, which confirms our previous finding about the relevance of the clock-controlled gene *DBP* to AAT.

### Master Regulators as Drug Targets

Recently omics technologies and computational approaches have become intriguing tools and approaches for drug discovery, making use of gene expression data. Exploiting the systems biology approaches in several studies [39,40,41,42], master regulators have been reported as potential therapeutic targets. By definition, master regulators are molecules that are located at the top of the hierarchy involved in the transcriptomic regulation, where the nodes tend to converge after certain upstream steps [43]. In biological processes, master regulators specifically regulate the expression of downstream genes either directly or through cascades thereby leading to altered phenotypes. In cellular context, dendritic cells involved in antigen-specific responses are regarded as the master regulators which serve as a major link between the innate and the adaptive immune system [44]. A recent study from Vargas et al on Alzheimer’s disease has proposed several therapeutic molecular targets for drug development based on master regulator analysis [45]. Similar analyses revealed potential drug targets experimentally for anaplastic thyroid carcinoma in which few transcription factors were proposed as master regulators [46]. Few aforementioned examples demonstrate that transcription regulatory networks and master regulators could be promising drug candidates analysed for investigating complex diseases (including Alzheimer’s disease and cancer) as they could be crucial drivers of the molecular mechanism of disease processes.

## 2. Materials and Methods

**Figure 1:**
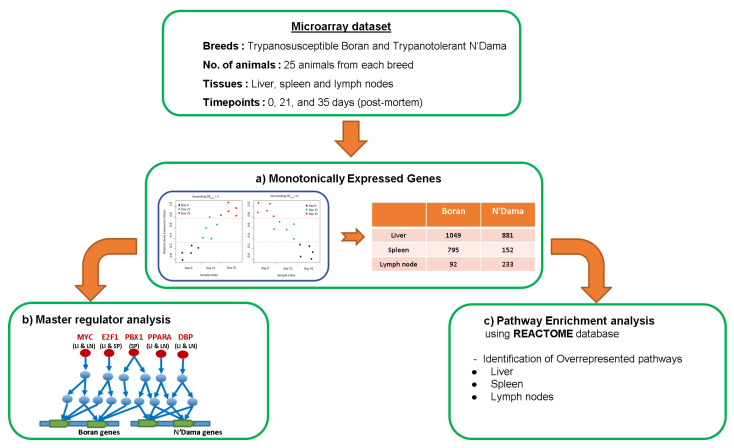
Flowchart of the analysis. (**a**) Monotonically Expressed Genes (MEGs) obtained from the analysis of microarray data set comprising gene expression profiles of two cattle breeds: trypanosusceptible Boran and trypanotolerant N’Dama (Boran Asc - MEGs expressed in the ascending pattern for Boran, Boran Des - MEGs expressed in the descending pattern for Boran, N’Dama Asc - MEGs expressed in the ascending pattern for N’Dama and N’Dama Des - MEGs expressed in the descending pattern for N’Dama; (**b**) Master regulator analysis (Red circles and texts in red represent the exemplarily selected master regulators from this study; LI, SP & LN stand for liver, spleen and lymph node tissues); (**c**) Pathway enrichment analysis performed using Reactome database.

In this section, we provided an outline of the data set and the methods we used in this study. Figure 1 depicts the workflow of this study.

### 2.1. Gene Sets

In order to identify master regulators and over-represented pathways related to the genetic programs underlying AAT, we analysed six gene sets that exhibit regular monotonic expression patterns in liver-, spleen-, and lymph node tissues of the two cattle breeds, namely trypanosusceptible Boran and trypanotolerant N’Dama, after being infected with *Trypanosoma congolense*. For this purpose, we took the gene sets from our previous study [27] in which we identified the genes based on the publicly available continuous transcription profiling time-series microarray data set (http://www.ebi.uk/arrayexpress/, accession no.E-MEXP-1778) [26]. In this study, we will mainly focus on the analysis of the gene sets. A brief summary about the microarray data set and the number of monotonically expressed genes (MEGs) is given below.

### 2.2. Microarray Data Set

In this section, we recapitulate the experimental procedure performed by Noyes et al. [26]. They performed a microarray experiment based on the cattle breeds Boran and N’Dama as per the following: In the animal experiment, 25 healthy trypanosomiasis-free animals from each breed (trypanosusceptible Boran and trypanotolerant N’Dama) were infected with *Trypanosoma congolense* IL1180 clone. To ensure the health of trypanosomiasis-free animals before experimental infection, the cattle were selected from herds in a tsetse fly-free and trypanosomiasis-free zone of the ILRI Kapiti Plains ranch and assessed negative for tick-borne parasites before transferring them to the ILRI research facility at Kabete. All procedures for handling the animals were performed according to the International Livestock Research Institute (ILRI) ethical review process. Liver, spleen and lymph node tissues were harvested from the cattle on day 0, day 21 and day 35. For control experiments, five animals from each breed were killed before infection and the gene expression readings were recorded for day 0. Tissue harvest was performed after infection. After infection of the cattle, the tissues were collected on day 21 and day 35 post-mortem. Additionally, needle biopsy method was applied only for the liver tissue sampling on day 0 (before infection), day 12, day 15, day 18, day 26, day 29, day 32 after infection. For each condition, extraction of RNA from tissues was done and hybridisation were performed on individual arrays.

### 2.3. Monotonically Expressed Genes

In our recent study [27], we identified the MEGs for each tissue of both cattle breeds by applying the monotonic feature selector (MFSelector) approach [47] to the microarray data set. The lists of MEGs for each tissue are provided in the Supplementary File S1 and the numbers of MEGs are given in Table 1.

### 2.4. Finding Master Regulators and Over-Represented Pathways

Similar to our previous studies [20,37,38], we applied well established systems biology approaches using the geneXplain platform [48] in order to identify master regulators and over-represented pathways.

For this purpose, we first run the “upstream analysis” workflow developed by [49] with the maximum radius of 10 steps upstream using the Reactome database [50]. The “upstream analysis” algorithm constructs a global signal transduction network and then identifies the master regulators based on the convergence points of these networks. In general, master regulators are located at the top of a regulatory hierarchy and control the downstream genes without their regulatory influence in signalling pathways [51].

Afterwards, we identified the over-represented pathways in order to unravel the functional properties of the MEGs. The knowledge about the over-represented pathways from Reactome database [50] provides mechanistic insight into the MEGs and helps to decipher novel biological functions underlying the AAT disease mechanisms.

## 3. Results

Mainly focusing on the regular monotonic changes of gene expression profiles in liver-, spleen-, and lymph node tissues during the AAT disease progression, we analyzed in this study for each tissue the related MEG set and identified master regulators as well as over-represented pathways.

### 3.1. Master Regulator Analysis

The “upstream analysis” workflow [48] has been employed using the MEG sets of the tissues in order to computationally identify a variety of master regulators. As a result, we obtained altogether 10 unique master regulators for both breeds across all tissues as shown in Table 2. Remarkably, the vast majority of the master regulators are highly distinct between trypanosusceptible Boran and trypanotolerant N’Dama breeds, only *PBX1* is found common for the spleen tissue of both breeds.

#### 3.1.1. Master Regulators in Liver

Using the “upstream analysis" workflow, we identified three master regulators (*MYC*, *E2F1*, *PPARG*) for the liver tissue of Boran and four master regulators (*DBP*, *PBX1*, *HOXA4*, *PPARA*) for N’Dama.

*MYC* is a member of the basic helix-loop-helix (bHLH) transcription factor family. It regulates a wide range of biological processes including metabolism, apoptosis, cell cycle, cell growth, angiogenesis or reprogramming in several tissues [52,53]. Importantly, *MYC* is highly pleiotropic [54] indicating that its deregulation is in close connection with all hematological malignancies, especially anaemia [55,56] which is a prominent feature of the AAT disease and also with drug resistance [56,57,58,59]. Furthermore, it has been reported as regulator of large networks of genes and has been associated with several cancer types, and is thus serving as a potential drug target [52,60,61,62]. Furthermore, it has been reported in the host-parasite interaction, improving the survival rate of parasites in surpassing immune surveillance mechanisms [63,64]. With regard to the parasite’s survival, MYC could be playing pivotal roles in induction and manipulation of host cell’s immunity in Animal African Trypanosomiasis as well. The master regulator *E2F1* found for Boran liver, plays a critical role in bile acid synthesis as per a study performed in mouse model [65]. *E2F1* inhibits the clearance of circulating cholesterol by regulating the expression of *PCSK9* [66], which might be related to the parasite’s critical need of cholesterol-related metabolism from Boran’s body, implicating the progressive conditions of hypocholesteraemia and hypolipidaemia after infection [67]. Peroxisome proliferator-activated receptor gamma (*PPARG*), found as the third master regulator for Boran liver, belongs to the nuclear hormone receptor super family [68,69]. In liver, induction of *PPARG* overexpression as a result of pathophysiological stress, has led to lipid accumulation. Interestingly, blocking of *PPARG* gene expression has reduced the accumulation of lipids and the expression of inflammatory genes [70,71]. Therefore, *PPARG* is strongly associated not only with the lipid metabolism but also with inflammatory processes [72]. This suggests the role of *PPARG* in the induction of lipid metabolism by *T. congolense* to utilise a high amount of energy from Boran, resulting in weight loss and loss of body conditions in the cattle during the AAT disease.

Albumin D site-binding protein (*DBP*), found for the liver tissue of N’Dama, is a liver-enriched transcription factor [73] and plays important roles in circadian rhythm in the mammals [30,74]. Specifically, it influences the circadian transcriptional regulation of several liver-specific genes namely P450 genes such as *Cyp2a4* and *Cyp2a5* [75,76]. Belonging to the PAR bZIP basic leucine zipper family, *DBP* accumulates following a stringent circadian rhythm in liver cells [30]. The circadian control of the liver gene *CYP7* encoding the cholesterol 7α-hydroxylase enzyme, which catalyses the metabolism of cholesterol to bile acids [29,77,78], establishes the strong indispensable association of the circadian rhythm and *DBP* in cholesterol homeostasis. Remarkably, the expression of *DBP* was found upregulated in a tolerant mouse model after *T. congolense* infection, suggesting the strong link of *DBP* and trypanotolerance [79]. Our findings are further well-supported by the results in [34], in which he established the association of the circadian rhythm with the infection of *T. congolense* in rodents. Strikingly, the master regulator *PPARA* (Peroxisome proliferator-activated receptor α) is a ligand-induced nuclear receptor that is highly expressed in the liver of mammals [80,81]. *PPARA* is well-known for its transcriptional regulatory role in metabolic and inflammatory pathways, making it a potential therapeutic target [82,83,84]. Particularly, it plays a crucial role in several metabolic processes, including bile and amino acid metabolism, transportation and metabolism of lipids, fatty acid beta-oxidation, ketogenesis and lipogenesis [81,85,86], which could contribute to the protection of the host from worsening conditions of AAT like weight loss and hypolipidemia.

Furthermore, *PBX1* (Pre-B-cell leukemia homeobox-1) is necessary for the maintenance of definitive hematopoiesis in the fetal liver, which indicates the host-protective role of *PBX1* from anaemia [87] and thus contributing to trypanotolerance of N’Dama. Another master regulator *HOXA4* from the homeobox family, is known for its role in hematopoiesis and B-cell progenitor population expansion [88], which implicates its importance in the production and maintenance of blood cells and immune cells, thus helping the cattle to control the major complications of AAT such as anaemia or parasitaemia.

#### 3.1.2. Master Regulators in Spleen

The analysis of MEGs for the spleen tissues of the cattle breeds reveals that *PBX1* is a common master regulator between Boran and N’Dama. It is essential for the spleen tissue-specific function of hematopoiesis [89]. Another key regulator, *E2F1*, found for the spleen tissue of Boran, has been reported as a suppressor of dendritic cell maturation [90], therefore implicating its role as a transcription factor for the immunosuppression in the infected cattle Boran. Macrophages and dendritic cells play a significant role in the innate immune system. In particular, they are involved in the production of interferon γ (IFN-γ), which is important for resistance against *T. congolense* [91]. Inhibition of dendritic cell maturation inhibits IFN-γ secretion [92], thus resulting in the reduction of the immune response against *T. congolense* [93]. The regulator *E2F1* in Boran spleen may have a leading role in immune depression of Boran, thus contributing to the susceptibility of Boran to AAT.

The remaining master regulator *PITX2*, found for the Boran spleen tissue, is a member of the bicoid transcription factor family, which is involved in a wide variety of developmental processes [94]. However, the reason for its importance and potential role with respect to host-pathogen interaction is still unclear.

#### 3.1.3. Master Regulators in Lymph Node

Investigation of the MEG sets of lymph node tissue unravelled three master regulators (*MYC*, p*STAT1*, and *PBX1*) for Boran and two master regulators (*DBP* and *PPARA*) for N’Dama. *MYC* plays an essential role in immune suppression and immune evasion mechanisms in assisting cancer cells to avoid the host’s immunity, as suggested in cancer studies [95,96]. It might play a role in helping the trypanosomes to escape the immune check points in host immune surveillance mechanisms, suggesting a major player in parasitaemia in Boran’s body which is one of the major characteristics of the AAT disease. Strikingly, we identified p*STAT1* (signal transducer and activator of transcription 1) as the second master regulator. The role of STAT1 is strongly associated with the development of Th1 and Th17 responses which are CD4+ T-cell subsets [97,98,99]. This mainly implicates overproduction of pro-inflammatory cytokines (like IL-17) leading to cell death and inflammation, which connects the severity of anaemia in Boran [100,101]. The master regulator *PBX1* has been studied in the homeostatic developmental programming of natural killer (NK) cells [102], which contributes to the main symptom of trypanosomiasis-associated acute anaemia as reported by [103].

*DBP*, identified as the master regulator in the lymph node of N’Dama, is a clock-controlled transcription factor and an important regulatory component of the circadian clock to ensure the 24 hour rhythm in mammalian species [104,105]. Several studies have reported the rhythmic expression of clock genes in cells of the immune system such as macrophages, dendritic cells and B-cells [106,107,108,109,110,111,112,113], representing the function of clock genes in immune responses. CD4+ T helper cells play crucial roles in the stimulation of effective antibody response and efficient isotype switching from IgM to IgG production [114,115,116], the critical features reported in N’Dama for its AAT tolerance [117]. These cells, being the significant regulators of adaptive immunity, harbor a circadian oscillator and generate cytokines such as IL-2, IL-4 and IFN-γ according to robust rhythms [118], implicating the tight connection of the circadian clock with adaptive immune responses during the AAT disease. Another interesting master regulator found for N’Dama is *PPARA* which has been reported to be expressed in B- and T-cells of the immune system [119,120]. Importantly, *PPARA* is known as the crucial regulator of immune responses such as inflammation and cytokine production [121,122,123]. According to its biological function in the immune system, *PPARA* could be protecting the depletion of the host’s cells from its own immune system during parasitic manipulation of immune responses, possibly controlling anaemia in N’Dama after trypanosome infection.

### 3.2. Pathway Analyses

**Figure 2:**
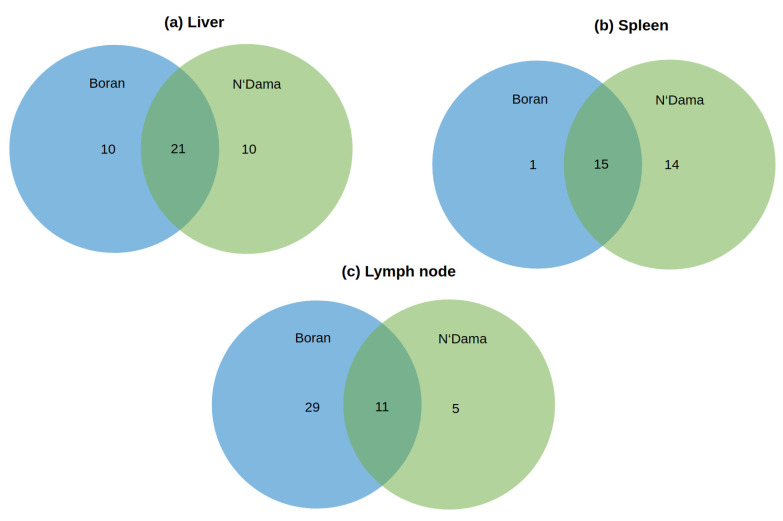
Venn diagram of over-represented pathways (*p*-adjusted <0.05) obtained for liver-, spleen- and lymph node tissues of the two cattle breeds Boran and N’Dama after the infection of *Trypanosoma congolense*. Pathway enrichment analysis was performed based on the Reactome pathway database [50].

To further decipher the specific biological functions of MEGs regarding AAT disease mechanisms, we investigated the over-represented pathways using the Reactome pathway database [50] for the three tissues of Boran and N’Dama. All the over-represented pathways obtained from the analysis are listed in the Supplementary File S2 and the pathways unique for each tissue of the two breeds are shown in Table 3, Table 4 and Table 5. Mainly focusing on these pathways, we found that several of them are overlapping between the breeds (see Figure 2a–c). Interestingly, Figure 2b shows that there is only one pathway unique for the spleen tissue of Boran. Taking the liver tissue into account (Figure 2a), there are only 10 unique pathways obtained for each breed despite the big overlap. On the other hand, remarkably high numbers of pathways are found for the lymph node tissue of Boran, in comparison to N’Dama (Figure 2c).

#### 3.2.1. Over-Represented Pathways Found for Liver Tissue

Analysis of over-represented pathways for the liver tissue of Boran and N’Dama uncovered ten pathways unique for each breed (see Table 3 and Figure 2a ).

**Figure 3:**
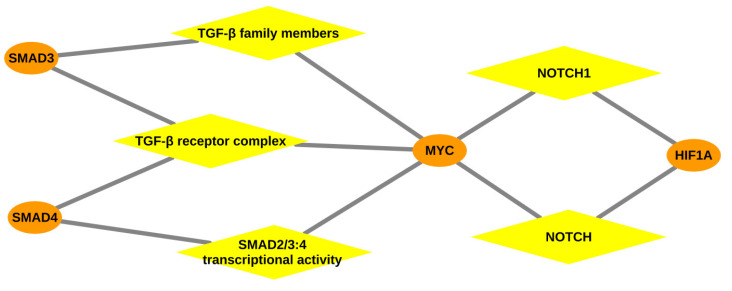
Gene-pathway network: Network representing the association between the over-represented pathways and transcription factors especially *MYC*, depicted for the liver tissue of Boran. Orange coloured ellipses in the network represent the transcription factor genes and yellow coloured rhombuses indicate the over-represented pathways. The transcription factors are connected to the pathways, thereby forming a network of interactions between them.

In Table 3, the top three over-represented pathways are associated with low oxygen environment and HIF accumulation due to parasitic infection [124,125] in the liver tissue of the trypanosusceptible breed Boran. Furthermore, the TGF-β signaling pathway, which includes the TGF-β receptor complex, TGF-β family members and SMAD2/SMAD3:SMAD4 heterotrimer (shown in Figure 3), is reported as a critically important pathway for the parasite in mammalian cell invasion and to escape the host’s immune system [126,127]. Especially, this pathway is involved in the suppression of macrophages that are essential players against parasites [128,129], implicating immunosuppression [130,131] during AAT in Boran. Moreover, our findings also lend support to experimental studies on TGF-β on other species of *Trypanosoma* [132,133]. Importantly, the interleukin-1 family signaling pathway found for Boran has been reported in direct association with damaging inflammation [134], which could explain the development of anaemia in Boran.

**Figure 4:**
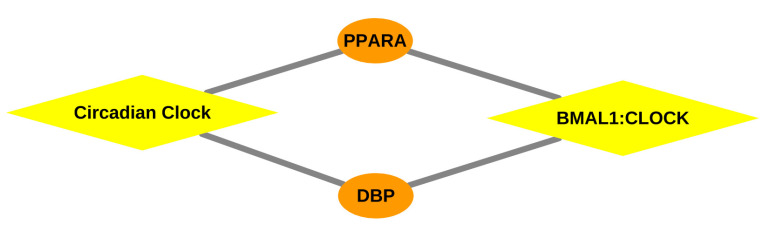
Gene-pathway network: Network representing the association between the over-represented pathways and transcription factors, especially *DBP*, depicted for the liver, spleen and lymph node tissue of N’Dama. Orange coloured ellipses in the network represent the transcription factor genes and yellow coloured rhombuses indicate the over-represented pathways. The transcription factors are connected to the pathways, thereby forming a network of interactions between them.

Regarding N’Dama’s liver tissue, the circadian clock and its components are inextricably in association with the transcriptional regulation of liver functions [135,136,137,138], suggesting the integrity of the circadian rhythm in the trypanotolerance mechanisms of N’Dama as shown in Table 3 and Figure 4. This finding is consistent with our previous results [27] and also a recent study by Solis et al. [31]. Intriguingly, the second and third over-represented pathways found for N’Dama are emphasizing the activation of HOX genes, which have been implicated as master regulator genes in the process of haematopoiesis [139]. Haematopoietic cells, derived mainly from fetal liver and bone marrow, are important in self-renewal and long-term supply of blood cells, especially T cells, which play a crucial role in the immune system [139,140,141,142]. These pathways strongly suggest their potential roles in protection of N’Dama from anaemia which is the most prominent feature of AAT disease.

#### 3.2.2. Over-Represented Pathways Found for Spleen Tissue

For the spleen tissue, we identified one unique significantly over-represented pathway for Boran and 14 significantly over-represented pathways for N’Dama (see Table 4 and Figure 2b).

The over-represented pathway found for Boran’s spleen tissue is related to the activation of genes during the proliferation by transcription factors *POU5F1*, *SOX2*, and *NANOG*. This pathway is also associated with the downstream processes related to self-renewal and pleuripotency in embryonic stem cells [143,144] which appears to be the normal function of spleen tissue in mammals.

Circadian clock related pathways in Table 4 can be categorised as the most prominent pathways in the spleen tissue due to the circadian control of splenic macrophages and B-cell development [107,145] (see Figure 4). In particular, these pathways are essential in the context of the trypanotolerance of N’Dama, since circadian regulation of immune responses are controlled at various levels in mammals [31,73]. Fundamentally, as reported in previous studies [22,23,26,146], MAPK family signalling cascades have been demonstrated to play critical roles in immune response through the production of pro-inflammatory cytokines in macrophages, suggesting their role in host defense against *T. congolense* in N’Dama [22,23,26,146]. Another striking pathway (in Table 4 and Figure 4) is related to the regulation of lipid metabolism by *PPARA*, which strongly establishes the relationship of spleen tissue with cholesterol metabolism in trypanotolerant N’Dama. Splenomegaly and hypocholesteraemia [67], being clinical signs of AAT, are indications of high workload of the reticuloendothelial system [147,148] for the parasite clearance in the blood. This pathway could be controlling the AAT conditions of splenomegaly and hypocholesteraemia in trypanotolerant N’Dama.

#### 3.2.3. Over-Represented Pathways Found for Lymph Node Tissue

We identified 29 and 5 unique significantly over-represented pathways for lymph node tissue of Boran and N’Dama, respectively(see Table 5 and Figure 2c).

**Figure 5:**
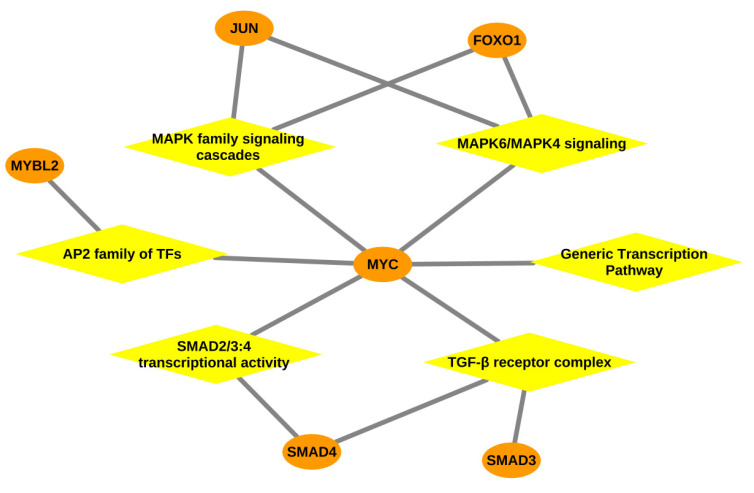
Gene-pathway network: Network representing the association between the over-represented pathways and transcription factors especially *MYC*, depicted for the lymph node tissue of Boran. Orange coloured ellipses in the network represent the transcription factor genes and yellow coloured rhombuses indicate the over-represented pathways. The transcription factors are connected to the pathways, thereby forming a network of interactions between them.

Inspection of Table 5 shows that we obtained a list of immune-related pathways for the lymph node tissue of Boran, suggesting the activation of immune cells in response to trypanosome infection. The toll-like receptor- and MyD88-related signalling pathways, being the major pathways for pathogen recognition in the innate immune system, have been reported as activated after protozoan infection [149]. Furthermore, activation of MAPK family signalling cascades, cytokine signalling, Fc epsilon RI signalling, interleukin-17 (IL-17) and interleukin-1 (IL-1) signalling strongly suggest that the inflammatory responses are following the cytokine production in response to *T. congolense* infection in Boran, as reported in previous studies [23,26,150]. Conversely, it seems quite possible that few pathways are under the manipulation of the parasite leading to the hyperproduction of proinflammatory cytokines resulting in catastrophic inflammation of host cells in Boran, especially involving *MYC* (see Figure 5). Particularly CD4+ T cells, which secrete IL-17, are reported in autoimmunity wherein IL-17 development is promoted by cytokines IL-1 and TGF-β [151]. The autoimmune phenomena of Boran result in the chronic destruction of own cells mainly leading to severe anaemia. Moreover, the major immune-related MAPK family signalling cascade has been demonstrated as the targeted pathway for manipulation by *T. congolense* in the host to escape the host immune responses [150].

For the lymph node tissue of N’Dama, two circadian clock related pathways have been found over-represented as shown in Table 5 and Figure 4, implicating the functioning of the immune system intimate accordance with the circadian clock as reported in several studies [31,33,152,153]. Important functional aspects of the immune responses such as phagocytosis, antigen presentation and immune regulation are regulated by the circadian clock [107], suggesting the circadian control of immunity against *T. congolense* infection in trypanotolerant N’Dama. Furthermore, interleukin-12 family signalling harbors IL-12, which is mainly leading to IFN-γ production which has been reported to be responsible for resistance to trypanosomiasis [154,155]. Remarkably, deletion of IL-12 in *T. b. brucei* and *T. evansi* infection models has resulted in the deficient IFN-γ production required for controlling parasitaemia [154], emphasizing the critical role of interleukin-12 family signalling in establishing resistance to AAT in N’Dama.

### 3.3. Analysis of Gene Expression Profiles of DBP and MYC

**Figure 6:**
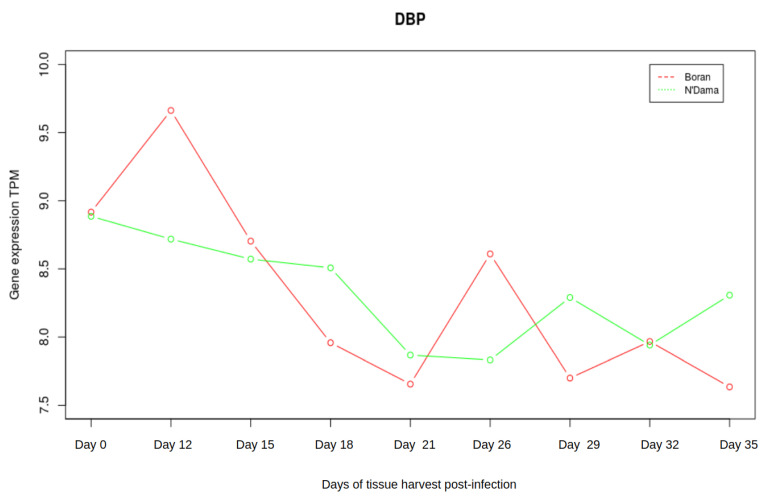
Expression of *DBP*. The red line in the plot corresponds to Boran whereas the green line corresponds to the breed N’Dama. In the early timepoints, expression of *DBP* reaches a peak in Boran and then starts to drop down. After the timepoint 5, the curve again increases slightly and at the later stages, it falls down again. On the other hand, the expression of *DBP* in N’Dama steadily drops until the timepoint 5, then it gradually increases.

**Figure 7:**
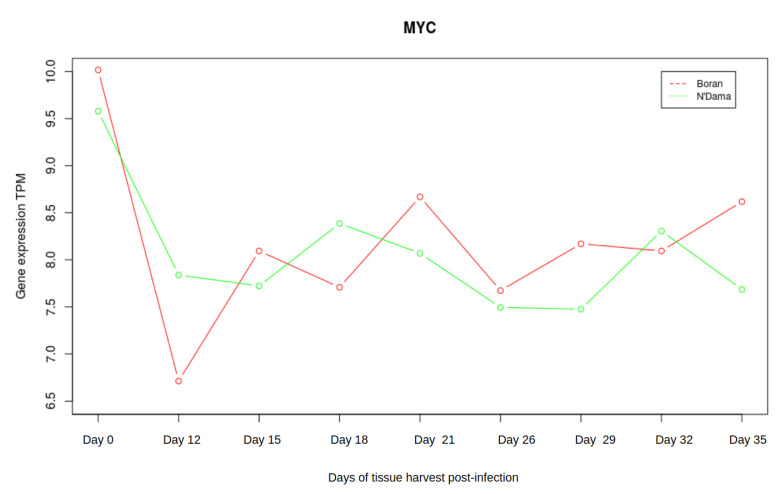
Expression of *MYC*.The red line in the plot corresponds to Boran whereas the green line corresponds to the breed N’Dama. In the early timepoints, expression of *MYC* decreases in both Boran and N’Dama. At the later stage, the expression of *MYC* increases for Boran whereas it decreases for N’Dama.

Taking the liver tissue into account, the microarray data set consisted of gene expression profiles for nine timepoints namely day 0, days 12, 15, 18, 21, 26, 29, 32, and 35. Emphasizing the crucial roles of the two master regulators *DBP* and *MYC*, we were interested in additionally investigating the changes in gene expression pattern of *DBP* and *MYC* of Boran and N’Dama during the disease progression. Interestingly, we observed slight antagonistic patterns of gene expression at different stages of the AAT disease, although both of these genes are not differentially expressed. The expression of *DBP* is abruptly declining for Boran from timepoint 2 (see Figure 6). Then, *DBP* increases its expression slightly and again declines towards the later stages. In contrast, the expression of *DBP* shows an increasing trend towards the later stages in N’Dama, despite the continuous steady decrease in its expression at earlier timepoints. It might be important to consider the expression of *DBP* during the later stages of the disease progression. AAT could be exhibiting similar effect in both cattle breeds during the earlier timepoints of infection. On the other hand, the expression of *MYC* seems quite clear for Boran with increasing tendency at the later timepoints (as shown in Figure 7). Whereas for N’Dama, *MYC* decreases its expression at several timepoints. These minor changes could be greatly contributing to susceptibility or tolerance mechanisms of both cattle breeds.

## 4. Discussion

African Animal Trypanosomiasis (AAT) is a vector-borne disease spread through the tsetse fly by carrying pathogenic African trypanosomes in its saliva. Clinical signs such as anaemia, hepatomegaly and splenomegaly are displayed in the cattle during the course of the AAT disease, which gradually progresses in multiple steps. Based on its signs, the AAT disease shows a multi-stage progression process in the body of the animal. Previous studies have pointed out that the consideration of monotonic expression patterns of genes (MEGs) could reflect the stage-by-stage progression of the disease [47,156]. Thus, we analysed several MEG sets in this study to identify master regulators which govern the transcriptional machinery of tissue-specific gene expression and thus influencing trypanosusceptibility and trypanotolerance of the breeds Boran and N’Dama, respectively. For this purpose, the consideration of the three tissues, liver, spleen, and lymph nodes are quintessential since they are the primary target sites of trypanosome infection. Inextricably, these tissues play crucial roles in generating host immune responses especially by increasing the number of macrophages, which results in the production of pro-inflammatory cytokines [35,146].

Remarkably, our findings suggest that the master regulators *DBP* and *MYC* identified for liver and lymph node tissues, appear to be greatly influencing the genetic programs for trypanosusceptibility and trypanotolerance mechanisms in Boran and N’Dama. Notably, *DBP* could be supporting the regulation of immune responses [27,31,73,153,157] beccause of its function in the circadian oscillatory mechanism [105] thereby establishing trypanotolerance in N’Dama. On the other hand, *MYC* has been reported to be responsible for the disruption of the circadian clock in cancer cells [158,159,160], elucidating the possibility of a dysfunctional circadian rhythm in Boran. Furthermore, *MYC* has gained its importance as it directly programs inflammation and immune suppression [96], which are constantly reported conditions in trypanosome-infected Boran.

Kupffer cells, the largest immune cell population of macrophages resident in the liver tissue, play a critical role in the mononuclear phagocytic system mounting the first line of immune response to foreign antigens [161]. Immune responses in the liver tissue depend on the activation state of macrophages [161,162]. M1 and M2 polarization of macrophages are known as two extremes in which M1 (classically activated) is characterised by expression of high pro-inflammatory cytokines and M2 (alternatively activated) by high anti-inflammatory cytokines [162,163]. Surprisingly, all three master regulators *MYC*, *E2F1* and *PPARG* identified for the liver tissue of Boran in this study, have been reported as the regulators necessary for M2-like polarization of macrophages [164], which could be an advantage for the trypanosomes to increase their survival inside the host’s body, and thereby contributing to enhanced parasitaemia in Boran.

In order to gain more mechanistic insights and to discover novel biological functions underlying the AAT disease progression of both breeds, the investigation of over-represented pathways based on the MEG sets of tissues is crucial. Based on pathway analysis, we obtained a number of over-represented pathways (see Supplementary File S2), several of which are in agreement with the results of previous studies [23,26,150] and are activated due to trypanosome infection in both breeds. Remarkably, the majority of these pathways were found to be common for both cattle breeds, while few of them are unique and breed-specific which could provide an important clue for distinguishing the biological processes controlling the mechanisms underlying trypanosusceptibility or trypanotolerance of the cattle breeds. Consequently, we focused in this study on outlining the potential roles of breed-specific unique pathways (see Table 3, Table 4 and Table 5) in association with the level of trypanotolerance in the respective cattle breeds. Although we reported the major immune-related pathways in Boran, these pathways could be leading to inflammation due to hyperproduction of pro-inflammatory cytokines in the host cells [165], thereby contributing to susceptibility of this breed. Despite the activation of immune signalling pathways, dysregulation causes the death of infected animals, especially dysregulated cytokine networks and overproduction of inflammatory cytokines (hallmark of African Trypanosomiasis) [146]. Contrarily, circadian clock related pathways (see Figure 4), interleukin-12 family signalling, regulation of lipid metabolism, and MAPK family signalling cascades might be properly regulated in N’Dama, indicating the underlying mechanism for trypanotolerance in N’Dama during the AAT disease. Especially, the pathways related to the circadian clock bolster our previous findings [27] in highlighting the important role of *DBP* and circadian rhythm in the coordination of the immune responses in trypanotolerant breed N’Dama.

Today it is well-known that the knowledge about master regulators is fundamental since they greatly control the TFs and the related genes [166,167]. Further, it is also pivotal to understand the regulatory network of TFs that cooperatively regulate a large number of genes during a disease process [168]. In our previous study [27], by analysing the promoter regions of the MEGs, we reported the importance of several TFs and their preferential partner choices elucidating their roles during the AAT disease progression. The consideration of TFs and their cooperations only provides the information regarding the first level of the transcriptional regulatory hierarchy [168]. However, for gaining a proper understanding of the disease progress in both breeds, it is still necessary to establish the hierarchy of the transcriptomic regulation in order to identify the master regulators [166,167,168]. Thus, our main objective in this study was to identify the master regulators together with signal transduction pathways associated with the AAT disease as potential drug targets, to complement our previous study [27]. Our current study provides a combined knowledge along with our previous findings. On one hand, it evidently indicates that *DBP* functions more as a master regulator of the circadian clock in peripheral tissues, supporting the trypanotolerance mechanisms in the cattle breed N’Dama. On the other hand, our analysis remarkably leads to the identification of novel master regulator *MYC* in association with the trypanosusceptibility mechanism of Boran.

Taken together, the systematic investigation of the upstream master regulators and over-represented pathways governing the regulatory mechanisms of the trypanotolerance level of two cattle breeds could provide novel mechanistic insights into the tissue- and breed-specific genetic programs. In particular, we identified *MYC* and *DBP* (as represented in Figure 3, Figure 4 and Figure 5) as potential discriminators between the two cattle breeds, trypanosusceptible Boran and trypanotolerant N’Dama, which are likely to be promising therapeutic targets for future works and for the selective breeding of this trait.

## Figures and Tables

**Table 1:** Number of MEGs in ascending and descending order for liver-, spleen- and lymph node tissues for the cattle breeds Boran and N’Dama.

	Boran		N’Dama	
	Ascending	Descending	Ascending	Descending
Liver	741	308	757	124
Spleen	669	126	13	139
Lymph node	87	5	119	114

**Table 2:** Master regulators of the breeds Boran and N’Dama.

	Boran	N’Dama
**Liver**	*MYC*, *E2F1*, *PPARG*	*DBP*, *PBX1*, *HOXA4*, *PPARA*
**Spleen**	*PITX2*, *E2F1*, *PBX1*	*PBX1*
**Lymph node**	*MYC*, *pSTAT1*, *PBX1*	*DBP*, *PPARA*

**Table 3:** Significantly over-represented pathways found for the liver tissue of Boran and N’Dama (*p*-adjusted <0.05).

	Liver		
	Pathway Name	Hit Names	Adjusted *p*-Value
**Boran**	Cellular responses to external stimuli	Arnt, Fos, Hif1a, Hsf1	4.64 × 10${}^{-4}$
	Regulation of beta-cell development	Foxo1, Hnf4g, Nkx2.2	0.0032
	Regulation of Hypoxia-inducible Factor (HIF) by oxygen	Arnt, Hif1a	0.0033
	Cellular response to hypoxia	Arnt, Hif1a	0.0033
	Signaling by TGF-beta Receptor Complex	Myc, Smad3, Smad4	0.0036
	Signaling by TGF-beta family members	Myc, Smad3, Smad4	0.0063
	Signaling by NOTCH1	Hif1a, Myc	0.0105
	Transcriptional activity of SMAD2/SMAD3:SMAD4 heterotrimer	Myc, Smad4	0.0147
	Signaling by NOTCH	Hif1a, Myc	0.0349
	Interleukin-1 family signaling	Nfkb1, Smad3	0.0371
	Cellular Senescence	Fos, Jun	0.0416
**N’Dama**	PTEN Regulation	Atf, Jun	0.0025
	Activation of HOX genes during differentiation	Hoxa4, Jun, Meis1	0.0036
	Activation of anterior HOX genes in hindbrain development during early embryogenesis	Hoxa4, Jun, Meis1	0.0036
	BMAL1:CLOCK, NPAS2 activates circadian gene expression	Dbp, Ppara	0.0161
	PIP3 activates AKT signaling	Atf, Jun	0.0229
	Transcriptional regulation of pluripotent stem cells	Pbx1, Pou5f1	0.0229
	Intracellular signaling by second messengers	Atf2, Jun	0.0280
	Transcriptional regulation by RUNX2	Sox9, Stat1	0.0364
	Transcriptional regulation of white adipocyte differentiation	Pparg, Rxra	0.0424
	Circadian Clock	Dbp, Ppara	0.0488

**Table 4:** Significantly over-represented pathways found for the spleen tissue of Boran and N’Dama (*p*-adjusted <0.05).

	Spleen		
	Pathway Name	Hit Names	Adjusted *p*-Value
**Boran**	POU5F1 (OCT4), SOX2, NANOG activate genes related to proliferation	Pou5f1, Stat3	0.0034
**N’Dama**	Oxidative Stress Induced Senescence	Fos, Jun	0.0033
	BMAL1:CLOCK NPAS2 activates circadian gene expression	Dbp, Ppara	0.0052
	MAPK6/MAPK4 signaling	Foxo1, Jun	0.0052
	Signaling by NOTCH3	Hes1, Pbx1	0.0052
	Cellular responses to stress	Fos, Hsf1, Jun	0.0062
	Fc epsilon receptor (FCERI) signaling	Fos, Jun	0.0067
	Cellular responses to external stimuli	Fos, Hsf1, Jun	0.0135
	MAPK family signaling cascades	Foxo1, Jun	0.0166
	Circadian Clock	Dbp, Ppara	0.0166
	Signaling by NOTCH	Hes1, Pbx1	0.0203
	Generic Transcription Pathway	E2f1, Hes1, Sox9, Stat1, Tead1	0.0243
	Cellular Senescence	Fos, Jun	0.0243
	RNA Polymerase II Transcription	E2f1, Hes1, Sox9, Stat1, Tead1	0.0350
	Regulation of lipid metabolism by Peroxisome proliferator-activated receptor alpha (PPARalpha)	PPara, Rxra	0.0432

**Table 5:** Significantly over-represented pathways found for the lymph node tissue of Boran and N’Dama (*p*-adjusted <0.05).

	Lymph Node		
	Pathway Name	Hit Names	Adjusted *p*-Value
**Boran**	MAP kinase activation in TLR cascade	Atf1, Atf2, Fos, Jun, Nfkb1	2.37$\times 10^{-8}$
	Interleukin-17 signaling	Atf1, Atf2, Fos, Jun, Nfkb1	1.83 × 10${}^{-7}$
	MAPK targets/ Nuclear events mediated by MAP kinases	Atf1, Atf2, Fos, Jun	3.66 × 10${}^{-7}$
	MyD88 cascade initiated on plasma membrane	Atf1, Atf2, Fos, Jun, Nfkb1	5.54 × 10${}^{-7}$
	MyD88 dependent cascade initiated on endosome	Atf1, Atf2, Fos, Jun, Nfkb1	7.62 × 10${}^{-7}$
	MyD88:Mal cascade initiated on plasma membrane	Atf1, Atf2, Fos, Jun, Nfkb1	1.03 × 10${}^{-6}$
	MyD88-independent TLR4 cascade	Atf1, Atf2, Fos, Jun, Nfkb1	2.27 × 10${}^{-6}$
	Toll Like Receptor 3 (TLR3) Cascade	Atf1, Atf2, Fos, Jun, Nfkb1	2.88 × 10${}^{-6}$
	Toll-Like Receptors Cascades	Atf1, Atf2, Fos, Jun, Nfkb1	4.39 × 10${}^{-5}$
	MAPK6/MAPK4 signaling	Foxo1, Jun, Myc	4.66 × 10${}^{-4}$
	Innate Immune System	Atf1, Atf2, Fos, Jun, Ltf, Nfkb1	9.27 × 10${}^{-4}$
	Signaling by Interleukins	Atf1, Atf2, Fos, Jun, Nfkb1, Stat1, Stat3	0.0010
	PTEN Regulation	Atf2, Jun	0.0016
	MAPK family signaling cascades	Foxo1, Jun, Myc	0.0028
	Oxidative Stress Induced Senescence	Fos, Jun	0.0069
	Cytokine Signaling in Immune system	Atf1, Atf2, Fos, Jun, Nfkb1, Stat1, Stat3	0.0081
	Fc epsilon receptor (FCERI) signaling	Fos, Jun	0.0138
	PIP3 activates AKT signaling	Atf2, Jun	0.0154
	Transcriptional activity of SMAD2/SMAD3:SMAD4 heterotrimer	Myc, Smad4	0.0171
	NGF signalling via TRKA from the plasma membrane	Atf1, Stat3	0.0171
	Intracellular signaling by second messengers	Atf2, Jun	0.0189
	Immune System	Atf1, Atf2, Fos, Jun, Ltf, Nfkb1, Relb, Stat1, Stat3	0.0241
	Transcriptional regulation by the AP-2 (TFAP2) family of transcription factors	Mybl2, Myc	0.0311
	Generic Transcription Pathway	E2f1, Mybl2, Myc, Smad4, Sox9, Stat1	0.0338
	Mitotic G2-G2/M phases	Foxm1, Mybl2	0.0381
	Mitotic G1-G1/S phases	E2f1, Mybl2	0.0381
	Interleukin-1 family signaling	Nfkb1, Stat3	0.0430
	Signaling by TGF-beta Receptor Complex	Myc, Smad4	0.0456
	Cellular Senescence	Fos, Jun	0.0482
**N’Dama**	POU5F1 (OCT4), SOX2, NANOG activate genes related to proliferation	Pou5f1, Stat3	0.0042
	BMAL1:CLOCK, NPAS2 activates circadian gene expression	Dbp, Ppara	0.0078
	Circadian Clock	Dbp, Ppara	0.0244
	Factors involved in megakaryocyte development and platelet production	Irf1, Irf2	0.0354
	Interleukin-12 family signaling	Stat1, Stat3	0.0375

## Supplementary Materials

The following are available online at https://www.mdpi.com/1422-0067/22/2/562/s1, File S1: Lists of Monotonically Expressed Genes obtained from the MFSelector approach, File S2: Lists of all over-represented pathways obtained for liver-, spleen- and lymph node tissues of Boran and N’Dama. The common pathways between the breeds are highlighted in red.
